# The combination of salt and drought benefits selective ion absorption and nutrient use efficiency of halophyte *Panicum antidotale*

**DOI:** 10.3389/fpls.2023.1091292

**Published:** 2023-04-21

**Authors:** Tabassum Hussain, Hina Asrar, Wensheng Zhang, Xiaojing Liu

**Affiliations:** ^1^ CAS Engineering Laboratory for Efficient Utilization of Saline Resources, Center for Agricultural Resources Research, Institute of Genetics and Developmental Biology, Chinese Academy of Sciences, Shijiazhuang, China; ^2^ Dr. M. Ajmal Khan Institute of Sustainable Halophyte Utilization, University of Karachi, Karachi, Pakistan

**Keywords:** water deficit, salt, combine stress, ion regulation, oxidative stress, anatomical adaptations

## Abstract

Soil salinity and water deficit often occur concurrently, but understanding their combined effects on plants’ ion regulation is limited. With aim to identify if introducing drought with salinity alleviates salt stress’s ionic effects, *Panicum antidotale* – a halophytic grass- was grown in the presence of single and combined stressors, i.e., drought and salt (low and high). Regulation of cations and anions along with the antioxidant capacity and modifications in leaf anatomy were investigated. Results showed a combination of low salt and drought minimally affected plant (dry) mass by improving the selective ions absorption and nutrient use efficiencies. The lowest ratio for efficiency of photosystem II and carbon assimilation (ΦPSII/ΦCO_2_) suggested less generation of reactive oxygen species, which were probably detoxified with constitutively performing antioxidant enzymes. In contrast, the combination of high salinity and drought escalated the adverse effects caused due to individual stressors. The selective ion absorption increased, but the non-selective ions transport caused an ionic imbalance indicating the highest ratio of Na^+^/K^+^. Although the area of mesophyll increased, a reduction in epidermis (cell number and area) predicted a mechanical injury prone to water loss in these plants. The compromised activity of antioxidant enzymes also suggested treatment-induced oxidative damage. Yet, the synergistic interaction between high salinity and drought was not detrimental to the survival of *P. antidotale.* Therefore, we suggest planting this grass in habitats with harsh environmental conditions to meet the increasing fodder demands without compromising agricultural lands’ productivity.

## Introduction

Rapid shift in global climatic conditions due to industrialization and urbanization has raised concerns like food and freshwater availability for a growing population (Islam and Karim, 2019). This situation is notably worse in the arid and semi-arid regions of the world, for instance, the food demand in Africa and Asia has exceeded the capacity of the food supply chain (Hussain et al., 2019; Fukase and Martin, 2020). Salinity and drought have affected 7% and 40% of the total land area, respectively, which makes these abiotic stressors as primary factors for agro-economic losses (Trenberth et al., 2014). The sustainable development goals were designed to overcome this distressing situation, which has united researchers across the globe to improve productivity under stress (Morton et al., 2019). Natural habitat often exhibited multiple stress factors, therefore, research needed to identify responses of plants under multiple stress conditions (Cominelli et al., 2013) to establish stress resilience of a plant.

Halophytes are the native flora of saline landscapes, of which many species also exhibit tolerance to drought conditions (Weber, 2009). Their utilization for phytoremediation, medicines, and biofuel has far surpassed their traditional uses as feed and fodder (Hamed and Custódio, 2019). The concurrence of stress factors, i.e., drought and soil salinity, is ubiquitous in natural habitats of the halophytes. Since many of the physiological and biochemical mechanisms adapted in response to these stressors are common, the exposure of halophytes to combined stressors may act additively, synergistically, or antagonistically to escalate their tolerance to stress environments (Hamed et al., 2013).

Excessive concentrations of Na^+^ and Cl^-^ limit the availability of other beneficial ions and disturb cellular osmotic potential. Conversely, plants under drought stress defend against water shortage by utilizing mainly K^+^ and Cl^-^ at a lesser extent for stomatal closure (Ruzin, 1999). The accumulation of Cl^-^, a common anion in response to both salt and drought stressors, benefits plants by improving the size of leaf cells (i.e., more water storage capacity) and by increasing the mesophyll conductance to atmospheric carbon (Colmenero-Flores et al., 2019). In addition, the oxidative stress generated either due to an enhanced ROS production or inhibited antioxidant activity is a typical response to salinity and water-deficit conditions (Miller et al., 2010).

Physiological water-deficit, common to salt and drought stress, restricts the cellular turgor. Reduced leaf area coupled with stomatal closure limits the availability of CO_2_ to chloroplasts and, therefore, the photosynthetic performance (Flexas et al., 2009; Osakabe et al., 2013). Although plants employ various anatomical adaptations, such as an increase in vascular tissues and thickness of cell walls, to overcome water shortage (Guerfel et al., 2009), this phenotypic plasticity has not been resolved concerning combined stresses.

*Panicum antidotale* Retz. is a cash crop halophyte that can survive up to seawater salinity (500 mM NaCl) concentration (Koyro et al., 2013; Hussain et al., 2015). Extensively distributed along with the marginal lands of Pakistan, this C4 perennial grass is utilized as a typical fodder. Detailed investigations have been conducted to characterize the physiological, molecular, and biochemical markers of salt tolerance in this fodder grass (Hussain et al., 2015; Hussain et al., 2021). Our previous work distinguished single and combined effects of drought and salinity stress on the gas exchange properties of this grass (Hussain et al., 2020). To our surprise, a combination of salinity and drought treatment ameliorated the plant’s performance by assisting water-retaining and carbon-fixing properties.

Salt-tolerant grasses are generally salt excluders (Munns, 2005). *P. antidotale* retains toxic ions within roots and hence, a lower concentration of Na^+^ and Cl^-^ in shoots (Hussain et al., 2015). However, an understanding of the management of toxic ions in response to combined stresses has not been developed for this grass. The current study was, therefore, designed to explore the ion homeostasis management in this species to a combination of stresses. We presume that better resilience of this grass to salinity + drought stress will be due to either restricted accumulation of toxic ions or anatomical adaptations that favor water and carbon supply. Following hypotheses were specifically tested: i) the exposure of plants to combined stressors will enhance the selective transport of toxic ion to shoots. ii) Plants will exhibit a more activated antioxidative machinery to tolerate combined stress. iii) Plants treated with combined stressors will develop a larger area of transport tissues.

## Materials and methods

### Plant growth and treatments

Mature seeds of *Panicum antidotale* were sown in perlite for germination in a growth chamber with a temperature of 25/15°C and day/night regimes of 14/10 hours (Abei, 1984). Seedlings at the three-leaf stage and with a size of about 8 cm were selected to transplant in pots (15 × 22 cm; 3 plants/pot and three pots for each treatment) filled with 4 kg quartz sand (1-2 mm diameter) and irrigated with half-strength Hoagland’s nutrient solution (Epstein, 1972). Seedlings were first acclimated to greenhouse conditions (28/16°C ±2 and 14/10 hours day/night regimes, 40-60% humidity, 600 ± 45 µmol Photon m^-2^ s^-1^ light) for two weeks. Salinity was introduced gradually (50 mM NaCl daily increment) until the desired concentrations, i.e., 100 and 300 mM NaCl, were attained. Similarly, the irrigation method gradually developed drought (30% field capacity) in 4-5 days. Thus, there were 6 treatments in total, i.e., control (C), 100 (LS), 300 (HS) mM NaCl (100% irrigation), drought (D), 100+D (LSD) and 300+D (HSD) (irrigated with 0, 100 and 300 mM NaCl at 30% field capacity). Salinity and drought conditions were maintained daily by supplying the lost water after weighing the pots. Nutrient solution was replaced every 3^rd^ day to continue the supply of nutrients. Pots were relocated randomly every 2^nd^ day to reduce the block effects in the greenhouse.

### Growth measurements

Plants were harvested after 30 days of stress treatments (salinity and drought) and separated into leaves, stems, and roots. The fresh weight of the leaf, stem, and roots was measured immediately. The number of leaves and height of the plant were recorded per pot. The dry weight of the leaf, stem, and root was determined after drying the plant samples at 70°C in an oven for 72 hours (until a constant weight was recorded).

### Anatomical parameters

Leaf from the 3^rd^ and 4^th^ nodes from the top of the shoot was selected, and the healthy middle portion of the lamina was excised for anatomical studies. Leaf samples were fixed in Carnoy’s fixative (v/v 60% ethanol + 30% chloroform and 10% glacial acetic acid) for 12 h and preserved in acetic alcohol solution (v/v 25% acetic acid and 75% ethanol). Gradual dehydration of samples was conducted with different grades of ethanol (from 75% to 100%), followed by washing with xylene. Leaves were then embedded in paraffin wax at 56-58°C, and transverse sections with 8 µm thickness were cut with a microtome. Permanent slides were made by a double staining method using fast green and safranin dyes (Ruzin, 1999). The images of sections were taken by a light microscope (Lecia DM5500B,Wetzlar, Germany) mounted with a digital camera (ToupCam TP605100A, China). Anatomical characteristics including total area, leaf width, number of epidermal layers (upper and lower), area of epidermal layers (upper and lower), area of the mesophyll region, area of the vascular bundle, and the ratio of the mesophyll and epidermal regions, were calculated by using ImageJ software.

### Measurement of ions in plant

Cations (Na^+^, K^+^, Mg^2+,^ and Ca^2+^) were measured in leaf, stem, and root samples with an atomic absorption spectrophotometer (Analyticjena ZEEnit 700 P, Germany). Dry plant material (0.2 g) was ground in a ball mill. Concentrated nitric acid was used for acid digestion in a microwave reaction system (Multiwave Pro – Anton Pear, GmbH, Germany) at 180 °C and further heat 150 °C for 90 min after adding 1 ml hypochlorous acid. Na^+^ and K^+^ contents were measured by emission spectrometry, while that of Mg^2+^ and Ca^2+^ were measured with absorption spectrometry. Ion ratios between Na^+/^K^+^, Na^+/^Ca^2+^, Na^+/^Mg^2+^ and Na^+/^(K^+^ + Mg^2+^ + Ca^2+^) were also calculated.

Anions (Cl^–^, $\text{S}{\text{O}}_{4}{\;}^{2}{\;}^{-}$ , and $\text{N}{\text{O}}_{3}{\;}^{-}$ ) were estimated by ion chromatography (Dionex ICS-2100, Thermo Scientific, USA). About 0.1 g (leaf, stem, and root) was extracted in di-ionized water at ~100 °C for at least three hours.

Selective absorption and selective transport of ion (K^+^, Mg^2+^, and Ca^2+^) over Na^+^ were estimated as follows.


$$\text{Selective absorption }=\;{\left[\text{ion}/\left(\text{ion }+\text{ N}{\text{a}}^{+}\right)\right]}_{\text{Root}}/{\left[\text{ion}/\left(\text{ion }+\text{ N}{\text{a}}^{+}\right)\right]}_{\text{Soil}.}$$

$$\text{Selective transport }=\;{\left[\text{ion}/\left(\text{ion }+\text{ N}{\text{a}}^{+}\right)\right]}_{\text{Leaf}}/{\left[\text{ion}/\left(\text{ion }+\text{ N}{\text{a}}^{+}\right)\right]}_{\text{Root}.}$$

Ion use efficiency (IonUE) of K^+^, Mg^2+^, and Ca^2+^ (KUE, MgUE, and CaUE, respectively) was estimated according to (Debez et al., 2010), i.e.

IonUE = leaf dry weight (g)/leaf ion content (g ion DW^-1^) for that particular ion.

### Carbon, nitrogen, and their ratios in leaf and root

Ball mill ground plant materials (leaf, stem, and root: about 10 mg) were analyzed for the content of nitrogen and carbon (Elementar, Germany). Data were expressed as a percent of dry weight, and the carbon-to-nitrogen ratio was calculated.

### Measurement of ΦPS2 and ΦCO_2_

Effective photochemical quantum yield of photosystem two (ΦPS2) and the quantum efficiency of CO_2_ assimilation (ΦCO2) was measured simultaneously on a matured fully emerged leaf (from 3^rd^ and 4^th^ node) at different PPFD regimens (from 0 to 2000 µmol photon m^-2^ s^-1^) by using an infrared gas analyzer, IRGA (LI6400XT, LI-COR Biosciences, Lincoln, NE, USA) equipped with a red-blue LED chamber with an area of 2 cm^2^ (6400-40, LI-COR Biosciences). Both parameters were plotted with the help of Sigma plot (v. 11) as a linear regression model for all treatments. We used the ratio of ΦPS2 and ΦCO_2_ as an oxidative stress indicator by pointing to the divergence in the electron transfer photochemistry (Fryer et al., 1998).

### Measurement of antioxidant capacities

Leaves were excised and immediately ground with the help of a mortar pestle in the presence of liquid nitrogen for protein extraction. Fine powder was mixed (1:10) with extraction buffer (50 mM potassium phosphate, pH 7.0) containing EDTA 1 mM, ascorbic acid 1 mM, PVPP 2% w/v, and triton X-100 0.5% (Gossett et al., 1994). The activity of antioxidant enzymes (catalase (CAT), ascorbate peroxidase (APx), guaiacol peroxidase (GPOx)) were performed on supernatant that was collected after centrifugation at 12,000 x *g* for 20 min at 4 °C. The activities of superoxide dismutase (SOD) and glutathione reductase (GR) were determined in protein extracted with the same extraction buffer but at pH 7.8. Bradford’s (1976) assay was performed to estimate proteins in these extracts. The activity of CAT was calculated as the decrease in H_2_O_2_ (25 mM) in 3 ml of reaction mixture containing 50 mM potassium phosphate buffer (pH 7.0) and 100µL plant extract during 1 min at 240 nm (ϵ = 39.9 mM^-1^ cm^-1^) (Abei, 1984). The activity of Apx was determined in 3 mL of reaction mixture (50 mM potassium phosphate buffer (pH 7.0), 0.2 mM EDTA, 0.5 mM ascorbic acid) and the absorption spectra (ϵ = 2.8 mM^-1^ cm^-1^) at 290 nm recorded after mixing 50 µL plant extract for 1 min (Nakano and Asada, 1981). The activity of GPOx (ϵ = 26.6 mM cm^-1^) was estimated after mixing 100 μL of plant extract in a reaction mixture containing potassium phosphate buffer (50 mM, pH 7.0), three mM H_2_O_2_, three mM guaiacol (Zaharieva et al., 1999). An increase in absorbance was recorded for 1 min at 470 nm. The activity of GR (ϵ = 6.2 mM cm^-1^) was calculated according to Foyer and Halliwell (1976). Decrease of absorbance at 340 nm during 1 min due to oxidation of NADPH by oxidized glutathione (GSSG) in the reaction mixture (100 mM potassium phosphate buffer (pH 7.8), 0.5 mM GSSG, 0.03 mM NADPH, five mM EDTA and 100 µL plant extract). The activity of SOD was determined based on the reduction of nitro blue tetrazolium (NBT) (Beyer Jr. and Fridovich, 1987). Reaction was initiated after adding 10 µL of 0.12 mM riboflavin with 1 ml reaction mixture (10 mM L-methionine, 0.05 mM nitro blue tetrazolium salt, and 0.75% Triton X-100 dissolved in 50 mM buffer have pH 7.8) and plant extract (40 µL). After 7 min, the absorbance at 560 nm was recorded. However, the absorbance of the formation of formazan without plant. Extract is considered 100%. The activity of SOD was expressed as percent inhibition of reaction per min.

All enzyme activities were recorded by UV-VIS spectrophotometer (UV-VIS 1750, Shimadzu, Japan) and expressed as units per mg protein (U mg^-1^ protein). Total antioxidant capacity (T-AOC) of *Panicum* under the said stress treatments was also measured by detecting the antioxidant capacity of substances to reduce Fe^+3^ to Fe^+2^ at 520 nm absorbance (Liu et al., 2018).

### Statistical analysis

The data is presented as mean ± SE (n = 3) for all parameters. Three-way analyses of variance (ANOVA) were performed to study the differences in parameters concerning salinity, drought, and organs (leaf, stem, and root: present in **Supplementary Material**). At the same time, a *post hoc* test (Bonferroni) was conducted to support the significance (P<0.05) difference among the means of each treatment. Statistical analyses were performed by IBM-SPSS (vr.24).

## Results

### Plant growth parameters

**Figure 1:**
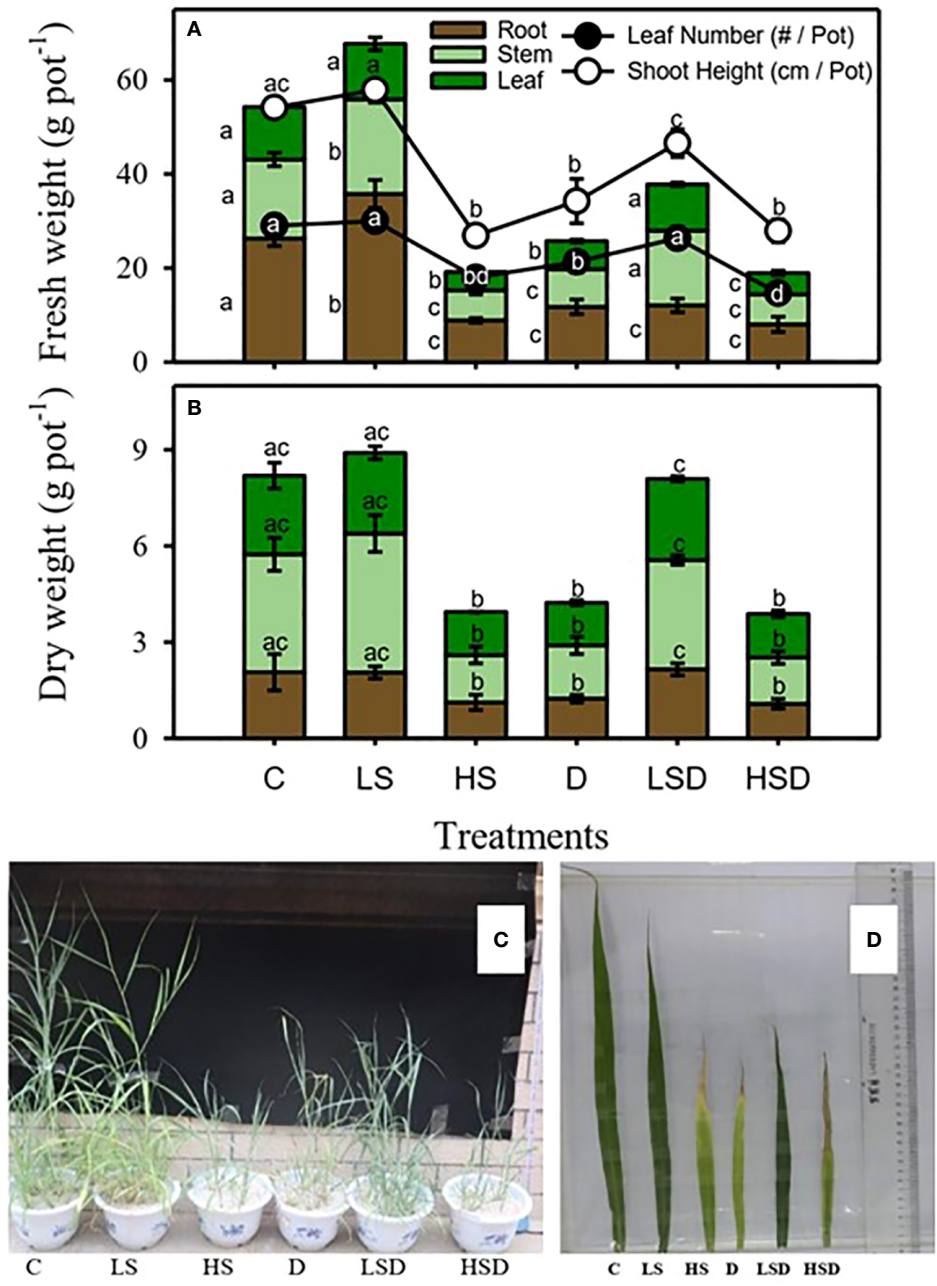
Biomass and growth of *P. antidotale* exposed to salt (0, 100, 300 mM NaCl abbreviated as C, LS, and HS respectively), drought (D), and combination of both (abbreviated as LSD and HSD) treatments. **(A)** fresh weight of leaf, stem, and root, leaf number, and shoot height. **(B)** dry weight of leaf, stem, and root. **(C)** plant growth and **(D)** leaf length. The values represent the means and ± S.E. (n = 3) where n is number of plants sampled from different pots. After the Bonferroni *post hoc* test, different letters denote significant difference at *P*< 0.05.

Changes in the biomass of *Panicum antidotale* under applied treatments, i.e., non-saline control (C), low salinity (LS: 100 mM NaCl), high salinity (HS: 300 mM NaCl), drought (D), low salinity combined with drought (LSD), and high salinity combined with drought (HSD), are displayed in **Figure 1**. Low salinity treatment significantly improved the fresh weight of stem, root and shoot height, but the fresh weight of leaf and leaf number remained unchanged compared to non-saline control plants. On the other hand, treatment with high salinity, drought, and combined stresses (LSD, HSD) caused significant inhibition in the biomass of roots, shoot height, and leaf number. However, the leaf number and fresh weight of stem and leaf at LSD were comparable to the control plant. In contrast, the HSD treatment significantly inhibited leaf biomass while shoot height and leaf number were like 300 mM NaCl and drought-treated plants. The dry weight of plants decreased two-fold under drought, high salinity, and HSD treatments.

### Carbon, nitrogen, and mineral regulations

**Figure 2:**
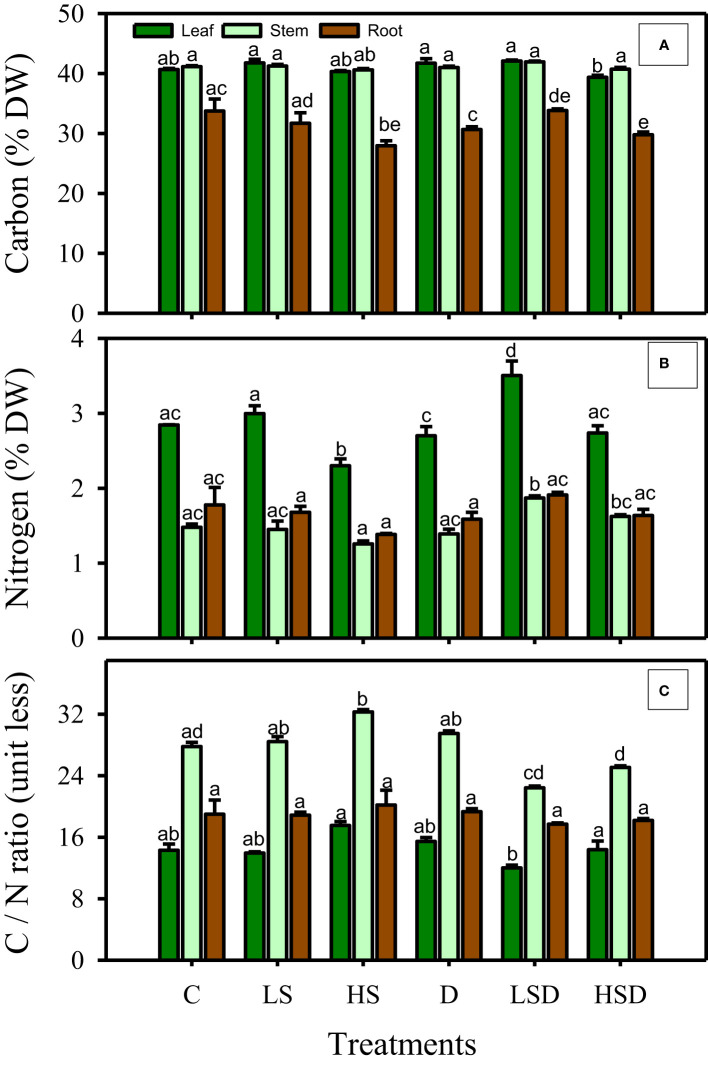
Percentages of carbon **(A)**, nitrogen **(B)**, and carbon to nitrogen ratios **(C)**, in leaf, stem and root under salt (0, 100, 300 mM NaCl abbreviated as C, LS and HS respectively), drought (D) and combination of both (abbreviated as LSD and HSD) treatments. The values represent the means and ± S.E (n = 3) where n is number of plants sampled from different pots. Different letters, after Bonferroni *post hoc* test, denote significant difference at *P*< 0.05.

The applied treatments did not affect leaf carbon, whereas leaf nitrogen was enhanced in response to LSD. However, the C/N ratio of all organs exhibited non-significant differences between the treatments (**Figure 2**).

**Figure 3:**
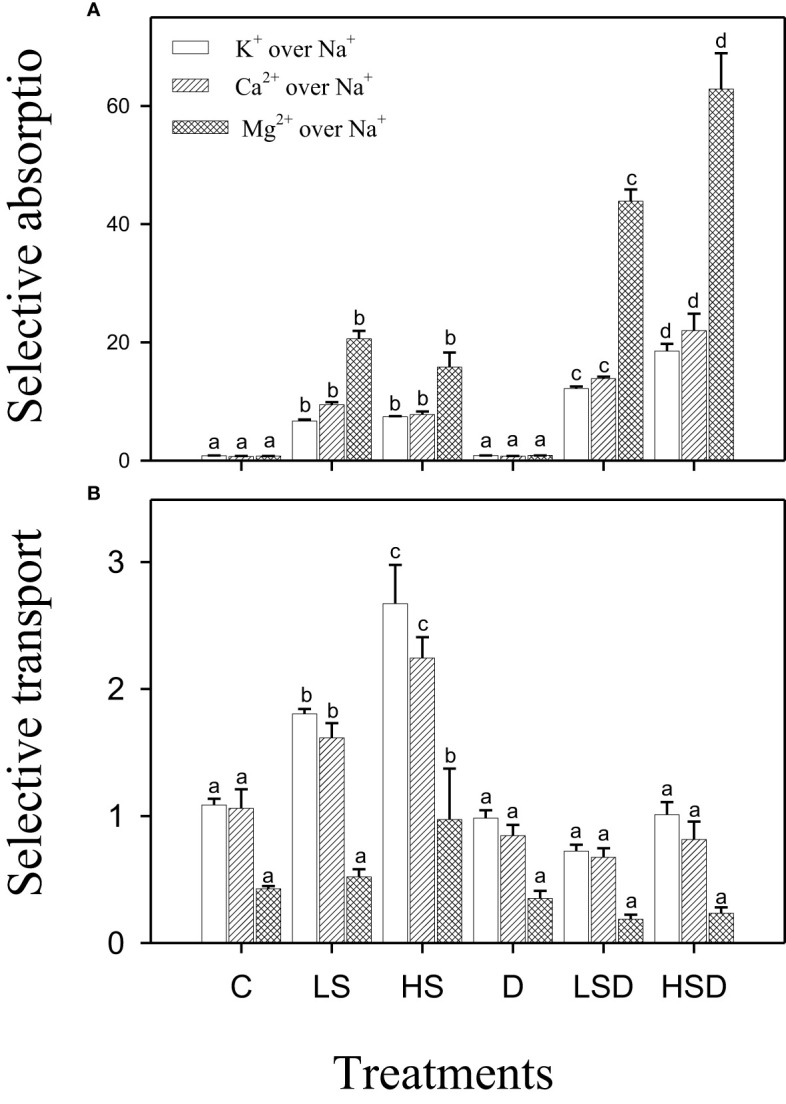
Selective absorption **(A)** and selective transport **(B)** of K^+^, Ca^2+^ and Mg^2+^ over Na^+^ under salt (0, 100, 300 mM NaCl; abbreviated as C, LS, and HS respectively), drought (D) and combination of both (abbreviated as LSD and HSD) treatments. The values represent the means and ± S. E (n = 3) where n is number of plants sampled from different pots. After the Bonferroni *post hoc* test, different letters denote significant difference at *P*< 0.05.

The selective absorption of beneficial ions, i.e., K^+^, Ca^2+^, and Mg^2+^, was increased in response to saline and combined stresses. It was positively related to NaCl concentration in the case of combined stress treatments. However, the selective transport of K^+^ and Ca^2+^ increased under saline treatments only, and a maximum increase was recorded at high salinity. On the other hand, the values for selective absorption of K^+^, Ca^2+^, and Mg^2+^ in plants treated with drought and combined stress were comparable to those in non-saline control. Interestingly, plants treated with HS also exhibited preferential transport of Mg^2+^ over Na^+^ in contrast to other treatments (**Figure 3**).

In the saline treatments alone, the highest accumulation of Na^+^ occurred in leaves, whereas, in the case of combined stress treatments, the maximum Na^+^ was noticed in the plant roots (**Table 1**). Conversely, the maximum accumulation of Cl^-^ occurred in leaves of combined stress-treated plants, while maximum Cl^-^ was recorded in the roots in response to salinity alone. The content of Na^+^ considerably increased in all the organs, i.e., root, stem, and leaf, under saline and combined stress treatments, the extent of which was positively related to NaCl concentration, except for the LSD treatment where root exhibited Na^+^ levels comparable to those in non-saline control plants. The accumulation of Cl^-^ followed a similar trend, but the leaf and stem of LS plants and the root of LSD showed Cl^-^ levels like that of control plants. The maximum content of K^+^ was recorded under non-saline and drought treatments. The exposure to NaCl, either alone or in combination with drought, lowered K^+^ accumulation in all the organs in a dose-dependent manner. Accordingly, all the organs exhibited the highest Na^+^ to K^+^ ratio in both HS and HSD treatments. HS treatment significantly reduced Ca^2+^ accumulation in plant leaves, whereas HSD inhibited its content in plant root. Interestingly, Na^+^ to Ca^2+^ ratio in root was comparatively less in combined stress than their counterpart saline treatments. The accumulation of Mg^2+^ in plant leaf and stem was not affected (p > 0.01) due to either salinity, drought, or salinity + drought treatments but decreased by 49% in root when plants were treated with HS, in contrast to non-saline control. Although the Na^+^ to Mg^2+^ ratio in leaf and stem was relatively high for combined stress, root for these treatments displayed a low ratio compared to their counterpart saline treatments. The contents of $\text{S}{\text{O}}_{4}{\;}^{2}{\;}^{-}$ in leaf and stem remained unchanged, but $\text{S}{\text{O}}_{4}{\;}^{2}{\;}^{-}$ in root was decreased by >20% in response to drought, salinity, and drought + salinity treatments. Accumulation of $\text{N}{\text{O}}_{3}{\;}^{-}$ in leaf was not affected with applied treatments but improved (57%) in stem with LSD and decreased with HS and HSD. The exposure of plant roots to drought treatment enhanced $\text{N}{\text{O}}_{3}{\;}^{-}$ accumulation, which did not change even under HSD. On the other hand, the plants treated with HS and LSD accumulated less $\text{N}{\text{O}}_{3}\;$ in the root.

**Table 1:** The effect of salt (0, 100, 300 mM NaCl; abbreviated as C, LS and HS respectively), drought (D) and combination of both (abbreviated as LSD and HSD) on cations (K^+^, Na^+^, Ca^2+^ and Mg^2+^), anions (Cl^-^, **$\text{S}{\text{O}}_{4}{\;}^{2}{\;}^{-}$** and **$\text{N}{\text{O}}_{3}{\;}^{-}$ **) and ratios between ions (Na^+/^K^+^, Na^+/^Mg^2+^, Na^+/^Ca^2+^, Ca^2+/^Mg^2+^ and Na^+^/(K^+^ + Ca^2+^ + Mg^2^)) on leaf, stem and root of *Panicum antidotale*.

	Treatments	K^+^	Na^+^	Ca^2+^		Mg^2+^	Cl^-^	$\text{S}{\text{O}}_{4}{\;}^{2}{\;}^{-}$	$\text{N}{\text{O}}_{3}{\;}^{-}$	Na^+^/K^+^		Na^+^/Mg^2+^	Na^+^/Ca^2+^	Ca^2+^/Mg^2+^	Na^+/^ (K^+^+Ca^2+^+Mg^+^)
		(mmol/Kg DW)													
**Leaf**	**C**	1130.3a	400.6a		372.5 a	115.0 a	479.0a	36.4a	32.0a	0.36a	3.49a		1.08a	3.25a	0.25a
	**LS**	752.7b	773.7b		336.4ac	86.7 a	490.8a	28.1a	25.8a	1.03b	9.04b		2.32b	3.90b	0.66b
	**HS**	461.6c	1308.0c		170.6 b	63.1 a	1186.1b	24.0a	21.2a	2.89c	21.85c		7.70c	2.79cd	1.88c
	**D**	761.7b	366.7a		254.5bc	90.1 a	451.2a	38.2a	24.2a	0.49ab	4.08a		1.45ab	2.85c	0.33ab
	**LSD**	525.7c	1074.3d		223.5 b	89.9 a	850.6c	27.4a	37.8a	2.05d	12.70b		4.98d	2.52cd	1.28d
	**HSD**	530.2c	1625.5e		212.7 b	87.0 a	1435.1d	28.0a	24.8a	3.13c	19.01c		7.74c	2.46d	1.96c
**Stem**	**C**	799.9a	221.4a		45.7 a	79.4 a	427.2a	40.5a	123.0a	0.28a	2.80a		4.87a	0.58a	0.24a
	**LS**	625.4b	544.9b		45.8 a	75.0 a	548.7a	30.6a	105.7a	0.88b	7.35b		11.96b	0.61a	0.73b
	**HS**	341.6c	863.3c		18.6 a	41.6 a	871.7bc	17.4a	30.6b	2.57c	20.82c		46.43c	0.45a	2.15c
	**D**	714.1a	283.6a		37.2 a	82.1 a	449.1a	43.2a	103.1a	0.41ab	3.41b		7.59b	0.45a	0.34a
	**LSD**	504.7d	896.1c		23.3 a	58.5 a	837.8b	16.8a	182.3c	1.77d	15.34c		39.57c	0.40a	1.53d
	**HSD**	348.6c	988.9c		17.1 a	44.4 a	1056.3c	21.7a	49.2b	2.85c	22.41d		65.46d	0.38a	2.41c
**Root**	**C**	591.5a	318.4ad		329.7 a	366.0 a	313.8a	164.2a	46.6ab	0.54a	0.88a		1.25a	0.84a	0.25a
	**LS**	430.6b	1393.4b		292.7 a	339.0ac	1216.3b	70.2b	59.1bd	3.24b	4.12b		4.79bd	0.87a	1.31b
	**HS**	293.6c	3022.7c		158.9 b	180.3 b	2098.1c	39.5b	26.7a	10.30c	17.89c		19.23c	0.96a	4.78c
	**D**	546.3a	238.1a		265.7ac	369.7 a	295.3a	52.0b	79.4cd	0.44a	0.65a		0.91a	0.72a	0.20a
	**LSD**	478.5b	476.9d		177.0 b	352.3ac	399.9a	65.8b	30.0a	1.00a	1.37d		2.69ab	0.51a	0.47a
	**HSD**	334.0c	1040.4e		184.8bc	300.1 c	1041.6	33.5b	61.6bd	3.14b	3.52b		5.85d	0.61a	1.27b

The values represent the means (n = 3) where n is number of plants sampled from different pots and different letters represent the difference between treatments at P<0.05 after Bonferroni *post hoc* Test.

The utilization efficiency of K^+^ i.e., KUE, and of Ca^2+^, i.e., CaUE, was improved in at least one of the organs in response to LSD treatment. For instance, the increment for KUE was noticed in the leaf and that for CaUE in the stem of *P. antidotale*. The utilization efficiencies of Mg^2+^ were like control in all the organs of these plants (**Table 2**).

**Table 2:** The effect of salt (0, 100, 300 mM NaCl; abbreviated as C, LS, and HS respectively), drought (D) and combination of both (abbreviated as LSD and HSD) on ion use efficiencies of K^+^, Ca^2+^ and Mg^2+^ (KUE, CaUE and MgUE respectively).

	Treatments	KUE		CaUE		MgUE	
Leaf	C	0.056ab	± 0.008	0.167a	± 0.035	0.888ab	± 0.172
	LS	0.087ac	± 0.011	0.188a	± 0.017	1.218a	± 0.159
	HS	0.075a	± 0.005	0.196a	± 0.003	0.906ab	± 0.117
	D	0.045b	± 0.002	0.131a	± 0.014	0.623b	± 0.098
	LSD	0.124c	± 0.006	0.294a	± 0.042	1.236a	± 0.213
	HSD	0.067ab	± 0.011	0.163a	± 0.024	0.664b	± 0.110
Stem	C	0.117a	± 0.016	2.006ab	± 0.262	1.912ad	± 0.278
	LS	0.178b	± 0.026	2.385a	± 0.309	2.433b	± 0.414
	HS	0.115a	± 0.027	1.976ab	± 0.314	1.485ae	± 0.298
	D	0.059c	± 0.007	1.262b	± 0.363	0.863c	± 0.189
	LSD	0.173b	± 0.005	3.780c	± 0.543	2.407bd	± 0.147
	HSD	0.106a	± 0.015	2.507a	± 0.939	1.355e	± 0.225
Root	C	0.089ab	± 0.024	0.170a	± 0.022	0.222a	± 0.035
	LS	0.122a	± 0.009	0.179a	± 0.028	0.249a	± 0.021
	HS	0.098ab	± 0.021	0.174a	± 0.030	0.271a	± 0.064
	D	0.059b	± 0.008	0.116a	± 0.010	0.137a	± 0.009
	LSD	0.115a	± 0.008	0.307a	± 0.042	0.256a	± 0.038
	HSD	0.084ab	± 0.014	0.154a	± 0.034	0.152a	± 0.030

The values represent the means and ± S. E (n = 3) where n is number of plants sampled from different pots and different letters will represent the difference between treatments at P<0.05 after Bonferroni *post hoc* Test.

### Antioxidative characteristics

**Figure 4:**
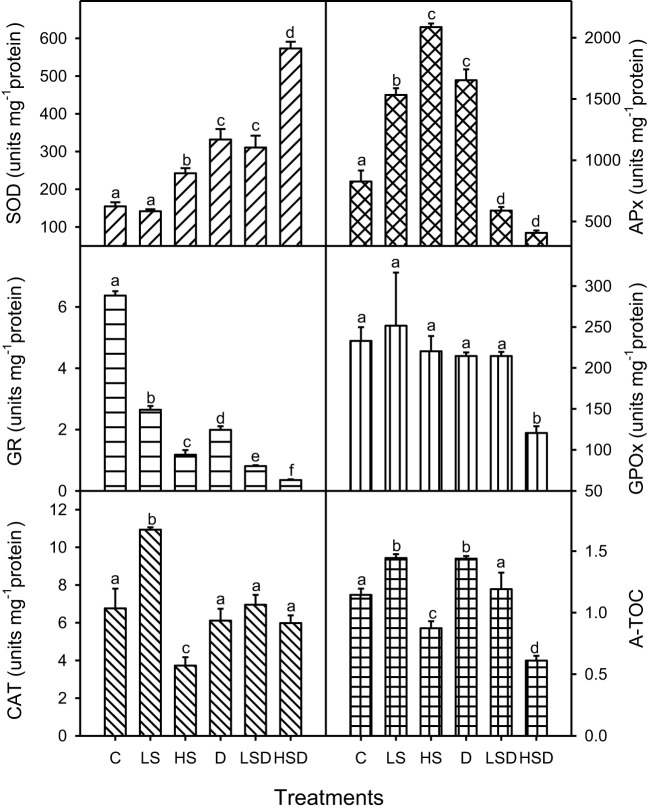
Antioxidant enzymes (SOD: superoxide dismutase, APx: ascorbate peroxidase, GR: glutathione reductase,GPOX: guaiacol peroxidase and CAT: catalase and total antioxidant capacity of *Panicum antidotale* under salt (0, 100, 300 mM NaCl; abbreviated as C, LS and HS respectively), drought (D) and combination of both (abbreviated as LSD and HSD). The values represent the means and ± S. E (n = 3) where n is number of plants sampled from different pots. After the Bonferroni *post hoc* test, different letters denote significant difference at *P*< 0.05.

Plant’s T-AOC was the highest under drought and low salinity (**Figure 4**). Although a reduction in this capacity occurred at LSD, the levels were comparable to non-saline control. In contrast, high salinity caused a significant reduction in A-TOC, and a further decrease was recorded at HSD. Analyses of antioxidant enzyme activities revealed a variable pattern under applied treatments. Such as, the maximum antioxidant activity of SOD was observed at HSD and that of APx under D and HS. Reduction in GPOx activity occurred only at HSD. While the activity of GR was inhibited by saline and drought treatments, the reduction was more pronounced under combined stresses, particularly at HSD. The activity of CAT prominently increased at low salinity and decreased at high salinity but was comparable to non-saline control and drought plants under combined stress treatments.

**Figure 5:**
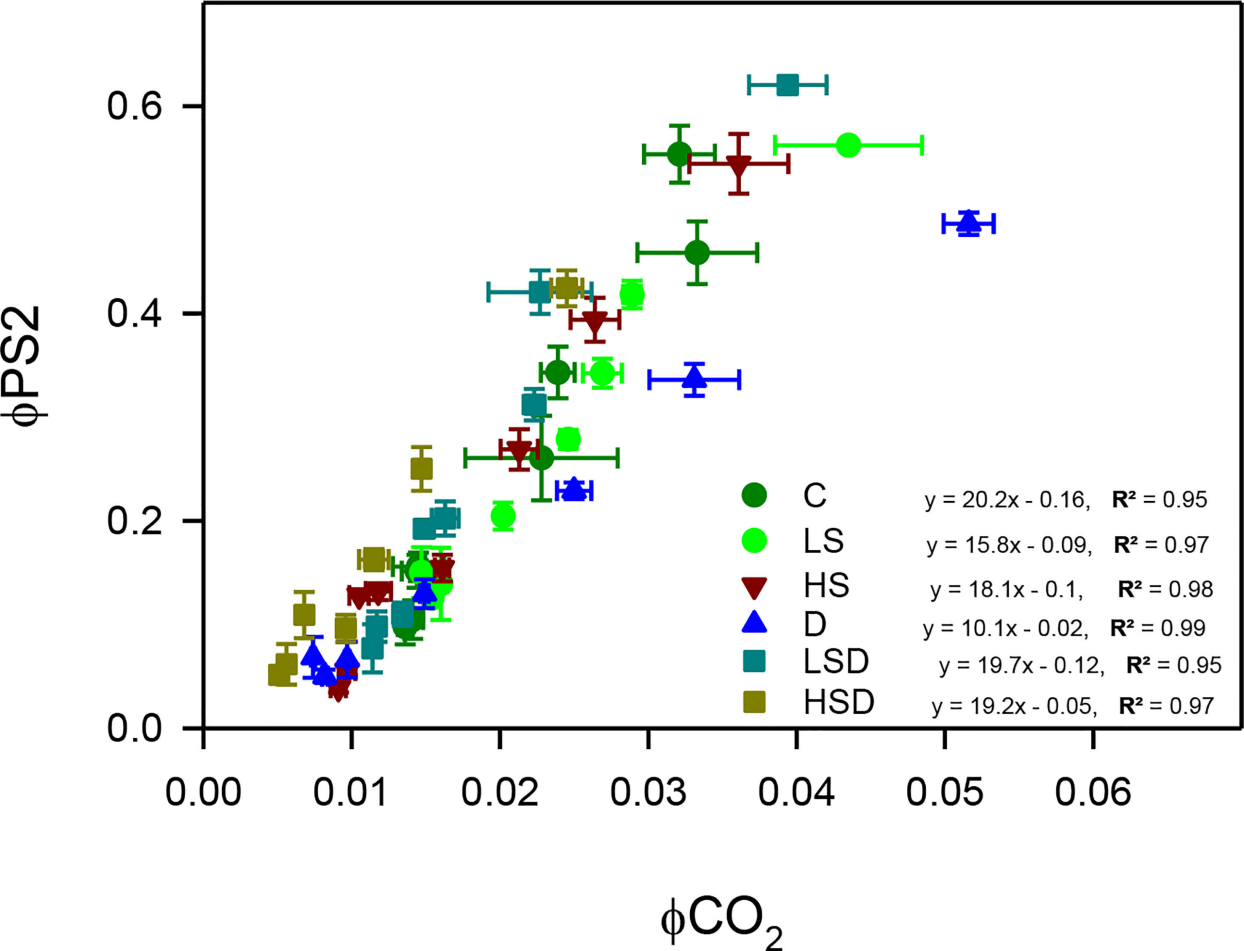
The ratio between quantum efficiencies of linear electron transport through PSII and of CO_2_ assimilation (ΦPSII/ΦCO_2_) of *Panicum antidotale* under salt (0, 100, 300 mM NaCl; abbreviated as C, LS, and HS respectively), drought (D) and combination of both (abbreviated as LSD and HSD). The values represent the means and ± S.E. (n = 3) where n is number of plants sampled from different pots.

Relation between PSII quantum efficiency and quantum yield for CO_2_ assimilation was analyzed (**Figure 5**). Results revealed that with any given PSII efficiency, the LS, HS, and D plants had a lower yield for CO_2_ assimilation. Interestingly, the lowest ΦPSII/ΦCO_2_ was displayed under combined stress, particularly at LSD.

### Anatomical responses

Plant leaf area and thickness significantly decreased under drought alone and with salinity (**Table 3**). The upper and lower epidermal cells decreased in number at D, HS, and a combination of both, i.e., HSD but the area of epidermal cells decreased only at HS. A significant reduction in leaf mesophyll area was recorded under high salinity, drought, and combined stress treatments, leading to a reduced mesophyll/epidermis ratio. The area of the vascular bundle also decreased in these plants with the minimum area of vascular bundle under drought.

**Table 3:** The effect of salt (0, 100, 300 mM NaCl; abbreviated as C, LS, and HS respectively), drought (D) and combination of both (abbreviated as LSD and HSD) on anatomical parameters of the leaf of *Panicum antidotale*.

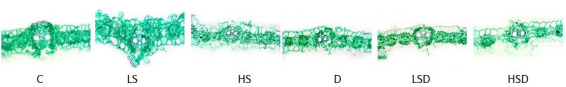										
Treatment	Total Area of the Leaf section (mm^2^)	Width of the leaf	Number of Epidermal cells (Upper)	Number of Epidermal cells (Lower)	Area of the Epidermal cells (Upper)	Area of the Epidermal cells (Lower)	Area of Epidermal cells	Area of the mesophyll region	RATIO MESO/EPI	Area of the Vascular Bundle
C	0.0697a	0.1347a	25.67a	25.67a	0.0120a	0.0127a	0.0247a	0.0313a	1.2853a	0.0137a
	± 0.0024	± 0.0037	± 1.20	± 1.67	± 0.0006	± 0.0009	± 0.0012	± 0.0015	± 0.0701	± 0.0007
LS	0.0730a	0.1523b	24.33a	24.67a	0.0117a	0.0147a	0.0263a	0.0330a	1.2543a	0.0137a
	± 0.0017	± 0.0032	± 0.33	± 0.88	± 0.0003	± 0.0012	± 0.0009	± 0.000	± 0.0204	± 0.0003
HS	0.0627b	0.1210c	18.00bc	15.33b	0.0183b	0.0183b	0.0367b	0.0147b	0.4060b	0.0113b
	± 0.0007	± 0.0051	± 2.08	± 0.88	± 0.0009	± 0.0009	± 0.0018	± 0.0018	± 0.0667	± 0.0007
D	0.0513c	0.1050d	19.33b	20.67c	0.0133a	0.0133a	0.0267a	0.0163bc	0.6213c	0.0083c
	± 0.0019	± 0.0021	± 0.33	± 0.33	± 0.0015	± 0.0009	± 0.0022	± 0.0003	± 0.0562	± 0.0003
LSD	0.0517c	0.1037d	24.33a	25.00a	0.0133a	0.0137a	0.0270a	0.0143b	0.5487bc	0.0103b
	± 0.0018	± 0.0009	± 0.33	± 0.58	± 0.0012	± 0.0013	± 0.0025	± 0.0017	± 0.1039	± 0.0003
HSD	0.0557c	0.1043d	15.67c	15.67b	0.0127a	0.0133a	0.0260a	0.0190c	0.7287c	0.0107b
	± 0.0027	± 0.0030	± 0.33	± 0.33	± 0.0007	± 0.0007	± 0.0012	± 0.0015	± 0.0290	± 0.0003

The values represent the means and ± S. E (n = 3) where n is number of plants sampled from different pots and different letters will represent the difference between treatments at P<0.05 after Bonferroni *post hoc* Test.

## Discussion

The present study investigated the effects of salinity (low and high), drought stress, and their combination on the management of ion homeostasis, antioxidant activity, and anatomical features of *P. antidotale*. These findings indicated that a combination of low salt and drought stress mostly improved plant performance by relieving plants from damaging effects of drought treatment. In contrast, the interaction between high salt and drought was mostly synergistic and escalated the harm caused by individual stressors. Thus, this halophytic grass can be naturally cultivated in low saline-arid areas to meet the increasing pressure on fodder and ecological needs.

Stimulated growth at low salinity (LS) and inhibited growth at high salinity (HS) of test species (**Figure 1**) are consistent with the response of other halophytic grasses (Flowers and Colmer, 2008). In addition, the equally reduced plant biomass at D and HS supports the similar effects of these conditions (Askari-Khorasgani et al., 2021). While a combination of low salinity and drought (LSD) alleviated the adverse effects of D on biomass accumulation (dry weight), the synergistic effects of high salinity and drought combination (HSD) mainly inhibited leaf biomass (fresh weight). The negative effects of HSD on plant growth and development were also evident with fewer upper and lower epidermal cells. However, the comparable leaf dry weight at HS, D, and HSD suggests similar influence of individual and combined stresses in impeding plants water acquisition. Although t reduction in epidermal cells was more pronounced at HS; the maximum area of these cells suggested a rigid outer leaf coat associated with epidermal cell wall-loosening proteins and is consistent with the protective and water-conserving function of the epidermis under stress (Zörb et al., 2015). The similar epidermal area in D and control plants highlights the regulatory adaptions that *P. antidotale* exhibits to overcome a water-deficit environment. It is worth mentioning that the drastic effects of D on roots’ fresh weight were not alleviated even with LSD. Since roots are the primary organs to perceive and respond to environmental stimuli, the altered expression of various genes may influence the development of the root system under stress (Cominelli et al., 2013). The compromised root growth, however, may be beneficial as it allows plants to redistribute the limited resources and fulfill the energy requirements of stress tolerance mechanisms (Munns and Gilliham, 2015).

Stress environments often cause a carbon/nitrogen imbalance shown with an increased C: N ratio suggesting the poor health (Rong et al., 2015) and discrepancies in the biophysical properties of plants (Xu et al., 2011). Significantly low C in the root of HS, D, and HSD-treated plants may reflect a disturbance in the transport of photosynthate from leaf or green stem to root. It also suggests a lower accumulation of C-based secondary metabolites, including soluble sugars, which function as osmolytes. Osmolytes have a promising role in maintaining the water influx while protecting the cells against structural and functional damage during stress (Jogawat, 2019). Interestingly, the unchanged nitrogen (N) content in root of HS, D, and HSD, reflected a non-disturbed nitrogen metabolism. *P. antidotale* treated with LSD showed the lowest C: N ratio in the leaves owing to the highest N and C content which implies abundant chlorophyll (Xu et al., 2018) and maximum carboxylation rates of Rubisco to sustain the photosynthetic performance of plants (Evans and Clarke, 2019). However, these plants could not mitigate the negative effects of D on the mesophyll region. Besides, the diffusional resistance of CO_2_ to bundle sheath reduces the carbon fixation in C4 plants (Ellsworth and Cousins, 2016). Therefore, determining the contribution of these factors behind non-optimal photosynthetic rates is equally important.

The limited plant growth under stress is mainly due to disturbed water relations, ionic toxicity, and nutritional imbalance. As expected, plants exposed to D did not experience ionic stress (i.e., comparable levels of Na^+^ and Cl^-^ in control and D plants). Moreover, the maintained K^+^ levels in the root and stem to that of control plants (**Table 1**) indicated a high resilience of our test species against the water deficit conditions. The accumulation of Na^+^ increased in all the organs with increasing NaCl, but that of Cl^-^ was limited to root, at least in LS-treated plants, which indicates the amplified susceptibility of below-ground plant parts to ionic stress. Combining drought with low salinity (i.e., LSD) enabled plants to retain ion homeostasis (Na^+^/K^+^, Na^+^/K^+^ + Ca^2+^ + Mg^2+^) at least in the root. The non-selectively transported ions (in low concentrations) may act as cheap osmoticum in the above-ground plant parts to develop a water potential gradient and improve the carbon assimilation properties of *P. antidotale* (Hussain et al., 2020). The combination of high salinity and drought (HSD) acted additively, and excessive accumulation of Na^+^ and Cl^-^ restricted the availability of essential ions (K^+^, Ca^2+^, and Mg^2+^) even in the root. The elevated Na^+^ and Cl^-^ damage cellular machinery and biomolecules, and, therefore, must either be excluded, extruded, or compartmentalized (Roy and Chakraborty, 2014). This is why the cytosolic ratio of beneficial ions, such as K^+^, to Na^+^, rather than the actual content of Na^+^, is considered more relevant to achieve stress tolerance (Anschütz et al., 2014). Salt-resistant species successfully maintain ion homeostasis due to the coordinated functioning of various ion transporters and channels (Huang et al., 2020). For instance, the upregulated expression of *SOS1* has been shown to restrict Na^+^ loading in the transpiration stream while enhancing K^+^ uptake under NaCl stress (El Mahi et al., 2019). Halophytic grasses express a supreme resiliency against salt stress by effectively managing toxic ions between different organs (Munns, 2011; Shabala, 2013). The reduced vascular bundle at HS proposed disturbed ions transportation efficiency, in agreement with the previous reports (Arafa et al., 2009; Nassar et al., 2020). The onset of drought caused a further reduction in the vascular bundle area, indicating the severe effects of water-deficit environments on the anatomical features of this halophyte. However, combining salt with drought, i.e., LSD and HSD, alleviated the negative impacts of drought on the area of vascular tissues, but the area was still low if compared to salinity stressor alone (**Table 3**). Our results, disapprove the hypothesis, “Plants treated with combined stressors will develop a larger area of transport tissues.”

Based on the analyses of selective absorption and transport of beneficial ions, we, for the first time, demonstrated differences in the ion regulatory capability of *P. antidotale* under saline-drought conditions. Combined stress improved the plant’s selective absorption of K^+^, Ca^2+^, and Mg^2+^ over Na^+^; however, the selective transport of the absorbed ions did not occur (**Figure 3**). Our data rejected the hypothesis that “the exposure of plants to combined stressors will enhance the selective transport of toxic ions to shoots”. In contrast, relatively less selective absorption under salinity (LS and HS) was followed by an active selective transport of beneficial nutrients. This enabled salt-treated plants to transport K^+^ from roots to stems and leaves while retaining most of the Na^+^ in the roots. This observation is surprising as an increased allocation of potassium in the below-ground parts of *Desmostachya bipinnata* – a halophytic grass with C4 photosynthesis, disturbed ionic homeostasis in the above-ground parts (Adnan et al., 2021). In our study, the LS-treated plants also selectively transported other ions such as Ca^2+^ from root to leaf and $\text{N}{\text{O}}_{3}{\;}^{-}$ from root to stem. Organ-specific nutrient allocation is related to the involvement of specific nutrients in cellular processes such as membrane stabilization, stomatal opening, regulation of enzyme activity, etc. (Wu and Wang, 2012). Better nutrient utilization of plant at LSD (**Table 2**) allowed us to claim an economical and environmentally sustainable solution to cultivate saline arid lands.

Plants under environmental stress experience an imbalance in photochemical reactions and electron flow from PSII, which affects their carbon assimilation properties. The energized electrons in such a case generate reactive oxygen species (ROS) (Foyer, 2018). The lowest ratio of ΦPSII/ΦCO_2_ in LSD plants suggested less availability of free electrons and an optimal photosynthetic performance as previously reported (Hussain et al., 2020). Interestingly, the high values of ΦPSII/ΦCO_2_ obtained in response to individual treatments, i.e., LS, HS, and D, decreased under combined treatments, i.e., LSD and HSD. The improved mesophyll region in HSD-treated plants (**Table 3**), however, does not guarantee an enhanced carbon fixation as the contribution of non-stomatal factors in limiting carbon fixation in high salinity + drought-treated plants was acknowledged (Hussain et al., 2020). Similarly, the enhanced ROS generation at HSD may also be reasoned to fewer stomata and a small stomatal aperture (Hussain et al., 2020).

SOD, the first cellular enzyme for ROS detoxification, was active in high salinity and drought-stressed plants, agreeing with the report on salt tolerant plants (Al Hassan et al., 2017). A combination of these conditions, i.e., HSD, interacted additively to produce more superoxide radicals and therefore, an enhanced SOD activity (**Figure 5**) as noted for other halophytes (Lu et al., 2021). The activity of subsequent H_2_O_2_-detoxifying enzymes, however, either remained unchanged (catalase) or decreased (APx) at HSD. While H_2_O_2_ in low concentrations modulate many physiological processes, high concentrations may damage metabolic machinery (Petrov and Van Breusegem, 2012). These findings allowed us to postulate a H_2_O_2_-derived damage to cellular biopolymers such as proteins and lipids in HSD-treated plants. The HSD-induced oxidative stress was also perceived with low activities of other antioxidants such as GR and GPx and the total antioxidant capacity. The relatively low (APx, GR) or comparable (GPOx, CAT) activities at LSD, to that of control plants, agreed with the posit that ROS production was low in these plants. The findings of the study reject the hypothesis that “plants will have more activated antioxidants machinery to tolerate combined stress”. In contrast, high ROS-detoxification efficiency under the combination of abiotic stresses has been reported for other plants (Ramu et al., 2016; Lopez-Delacalle et al., 2021).

## Conclusion

**Figure 6:**
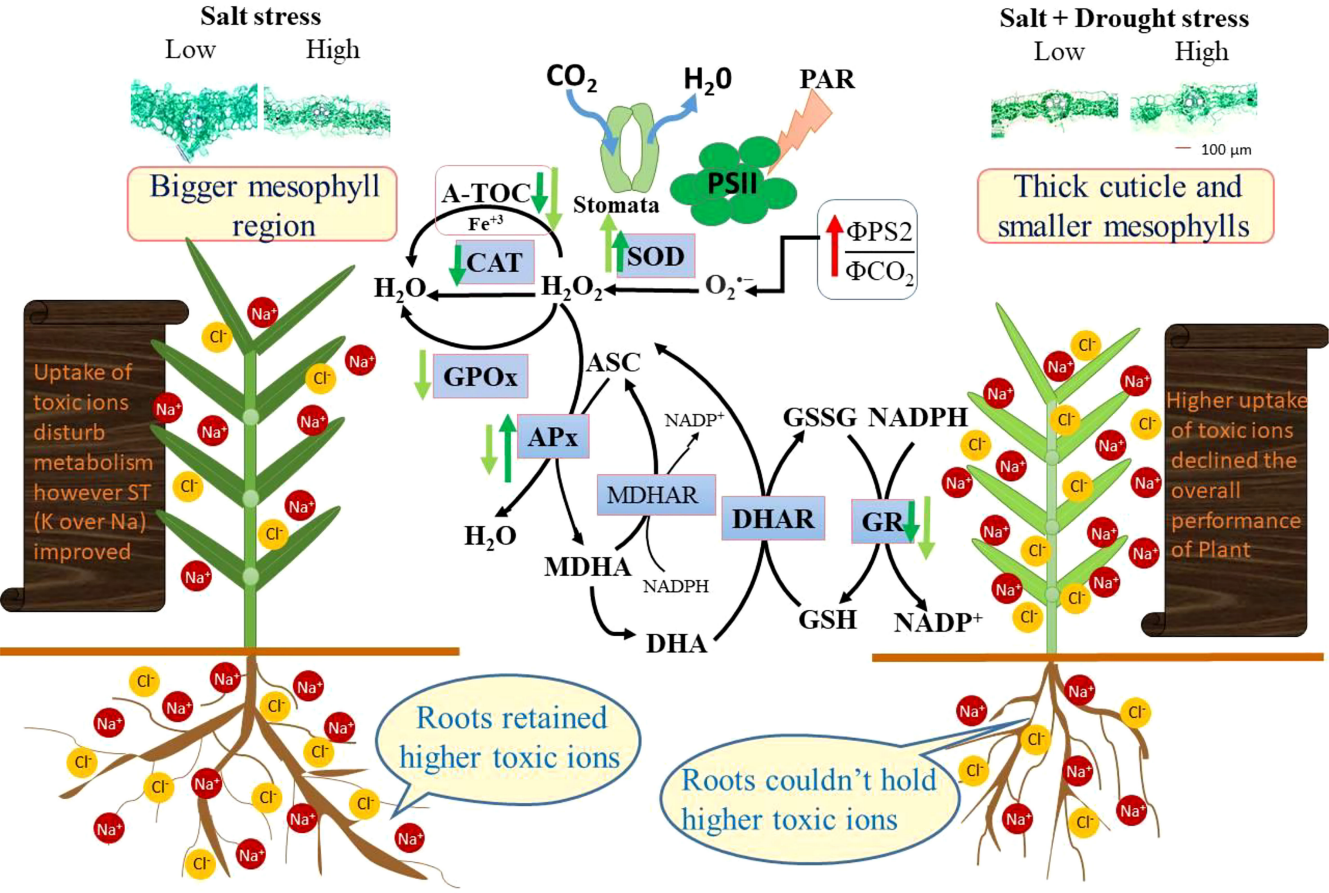
Schematic presentation of the individual (salinity) and combined (salinity + drought) stressors-induced alterations in ion regulation, antioxidant machinery and anatomical features of a halophytic grass *P. antidotale*. Arrows (up- or down-head) display an increase or decrease in a parameter’s response compared to control plants. The length of the arrow increases with increasing response difference. For each response, the arrow on the left stands for individual stress and on the right for combined stresses. SOD, superoxide dismutase; CAT, catalase; GPOx, glutathione peroxidase; APx, ascorbate peroxidase; DHA, ascorbate oxidized state; ASC, ascorbate reduced state; GSH, glutathione reduced state, GSSG, glutathione oxidized state; GR, glutathione reductase; ΦPSII/ΦCO_2_, the ratio between quantum efficiencies of electron transport and CO_2_ assimilation.

*Panicum antidotale* is a suitable candidate to grow on low saline-arid areas, as shown by dry weight accumulation (**Figure 6**). It was mainly due to the selective absorption of beneficial ions to fulfill the nutrient requirements and to utilize the accumulated ions as cheap osmoticum. In the absence of NaCl, drought-treated plants were able to accumulate high content of potassium, and this ability persisted after the addition of low salinity, i.e., LSD. Consequently, nutrient utilization efficiencies in these plants were improved compared with any of these two stressors individually (LS and D). Similarly, the production of ROS was minimal (i.e., ΦPSII/ΦCO2), and the T-AOC of test species was sufficient to detoxify the generated ROS. Nevertheless, the reduced area of the vascular bundle and mesophyll region explained many physiological strains in the current study (such as low photosynthesis and water content). Combining high salinity with drought (HSD) escalated the adverse effects caused by individual stressors on the antioxidant capacity and ion homeostasis. The non-selectively transported ions caused an ionic imbalance, and a high ratio of Na^+^/K^+^ was evident in all the organs. A reduction in epidermal tissues (number and area) predicted that these plants were prone to mechanical injury and water loss. Given all this, we would suggest highly antagonistic interactions between low salt and drought while synergistic/additive interactions between high salt and drought on most plant physiological mechanisms. Future studies must efficiently explore the molecular basis of combined tolerance to drought and salt stress to transfer tolerance traits in related crops.

## Data availability statement

The raw data supporting the conclusions of this article will be made available by the authors, without undue reservation.

## Author contributions

TH and XL perceived the idea. TH and WZ performed the experiments, TH evaluated data, TH and HA wrote the manuscript, WZ and XL corrected the manuscript. All authors contributed to the article and approved the submitted version.
