# Supplementary material for: The combination of salt and drought benefits selective ion absorption and nutrient use efficiency of halophyte Panicum antidotale
Source: Front Plant Sci. 2023 Apr 21;14:1091292. doi: 10.3389/fpls.2023.1091292 (PMC10160469; doi:10.3389/fpls.2023.1091292)
Supplement: Supplementary file 1 [file DataSheet_1.docx]

|  | NaCl | | Drought | | Organ | | NaCl x Drought | | NaCl x Organ | | Drought x Organ | | NaCl x Drought x Organ | |
| --- | --- | --- | --- | --- | --- | --- | --- | --- | --- | --- | --- | --- | --- | --- |
|  | *F* | *P* | *F* | *P* | *F* | *P* | *F* | *P* | *F* | *P* | *F* | *P* | *F* | *P* |
| FW | 120.4 | 0.001 | 119.2 | 0.001 | 80.6 | 0.001 | 29.3 | 0.001 | 6.9 | 0.001 | 30.7 | 0.001 | 10.3 | 0.001 |
| DW | 41.7 | 0.001 | 15.5 | 0.001 | 20.9 | 0.001 | 8.6 | 0.001 | 3.5 | 0.016 | 2.8 | 0.075 | 0.7 | 0.603 |
| N% | 19.4 | 0.001 | 17.9 | 0.001 | 322.0 | 0.001 | 6.3 | 0.005 | 8.7 | 0.001 | 1.2 | 0.313 | 2.8 | 0.040 |
| C% | 15.5 | 0.001 | 0.4 | 0.529 | 356.5 | 0.001 | 0.1 | 0.897 | 6.0 | 0.001 | 0.0 | 0.983 | 1.5 | 0.225 |
| δ15N | 28.7 | 0.001 | 9.7 | 0.004 | 96.1 | 0.001 | 5.1 | 0.011 | 1.6 | 0.187 | 2.5 | 0.097 | 1.3 | 0.299 |
| K^+^ | 196.2 | 0.001 | 24.4 | 0.001 | 87.1 | 0.001 | 15.5 | 0.001 | 6.9 | 0.001 | 12.8 | 0.001 | 5.6 | 0.001 |
| Na^+^ | 636.7 | 0.001 | 59.3 | 0.001 | 96.3 | 0.001 | 33.4 | 0.001 | 52.5 | 0.001 | 215.8 | 0.001 | 58.5 | 0.000 |
| Ca^2+^ | 14.3 | 0.001 | 8.9 | 0.005 | 108.8 | 0.001 | 5.4 | 0.009 | 2.1 | 0.105 | 1.3 | 0.290 | 1.2 | 0.345 |
| Mg^2+^ | 19.1 | 0.001 | 2.6 | 0.115 | 337.9 | 0.001 | 3.9 | 0.029 | 5.4 | 0.002 | 3.2 | 0.053 | 1.5 | 0.211 |
| Cl^-^ | 269.0 | 0.001 | 8.4 | 0.006 | 13.2 | 0.001 | 3.7 | 0.035 | 17.3 | 0.001 | 74.4 | 0.000 | 18.2 | 0.001 |
| SO_4_^2-^ | 5.9 | 0.006 | 2.7 | 0.111 | 10.7 | 0.001 | 1.8 | 0.186 | 1.6 | 0.193 | 2.6 | 0.089 | 2.2 | 0.093 |
| NO_3_^-^ | 21.2 | 0.001 | 7.0 | 0.012 | 67.4 | 0.001 | 1.3 | 0.276 | 14.8 | 0.001 | 1.6 | 0.214 | 7.3 | 0.001 |
| Na^+^/K^+^ | 534.0 | 0.001 | 63.5 | 0.001 | 119.9 | 0.001 | 59.6 | 0.001 | 67.4 | 0.001 | 161.5 | 0.001 | 53.7 | 0.001 |
| Na^+^/Mg^2+^ | 138.3 | 0.001 | 0.7 | 0.394 | 40.7 | 0.001 | 10.5 | 0.001 | 4.4 | 0.005 | 13.2 | 0.001 | 3.1 | 0.026 |
| Na^+^/Ca^2+^ | 50.2 | 0.001 | 4.8 | 0.035 | 76.2 | 0.001 | 2.1 | 0.141 | 18.2 | 0.001 | 12.0 | 0.001 | 2.4 | 0.071 |
| Ca^2+^/Mg^2+^ | 3.7 | 0.035 | 34.0 | 0.001 | 607.0 | 0.001 | 4.8 | 0.014 | 3.5 | 0.016 | 7.1 | 0.003 | 2.6 | 0.053 |

**Supplementary Table 1.** Three-way ANOVA on NaCl (0, 100 and 300 mM), drought and organs (leaf, stem and root) representing *F* and *P* values for fresh weight (FW), dry weight (DW), nitrogen content (N%), carbon content (C%), cations (K^+^, Na^+^, Ca^2+^ and Mg^2+^), anions (Cl^-^, SO_4_^2-^ and NO_3_^-^) and ratios between ions (Na^+^ / K^+^, Na^+^ / Mg^2+^, Na^+^ / Ca^2+^ and Ca^2+^ / Mg^2+^). The significant value was adjusted to 0.05.
